# Female Sexual Health: Barriers to Optimal Outcomes and a Roadmap for Improved Patient–Clinician Communications

**DOI:** 10.1089/jwh.2018.7352

**Published:** 2019-04-10

**Authors:** Sheryl A. Kingsberg, Jonathan Schaffir, Brooke M. Faught, JoAnn V. Pinkerton, Sharon J. Parish, Cheryl B. Iglesia, Jennifer Gudeman, Julie Krop, James A. Simon

**Affiliations:** ^1^ MacDonald Women's Hospital, University Hospitals Cleveland Medical Center, Cleveland, Ohio.; ^2^ Ohio State University College of Medicine, Columbus, Ohio.; ^3^ Women's Institute for Sexual Health, Nashville, Tennessee.; ^4^ University of Virginia Health System, Charlottesville, Virginia.; ^5^ Weill Cornell Medical College, White Plains, New York.; ^6^ MedStar Washington Hospital Center, Washington, District of Columbia.; ^7^ AMAG Pharmaceuticals, Waltham, Massachusetts.; ^8^ George Washington University, School of Medicine, IntimMedicine Specialists, Washington, District of Columbia.

**Keywords:** communication, education, menopause, sexual health, women

## Abstract

***Background:*** Although sexual health can be considered a vital sign for overall health, several barriers prevent women from receiving proper medical counseling, support, and/or care for their sexual health needs and concerns.

***Methods:*** Experts in sexual health compiled research and experience on the impediments to women receiving adequate assessment and treatment for their sexual health. Specific solutions and a roadmap for overcoming such barriers and improving patient–clinician communication are presented.

***Results:*** Social stigma around female sexuality remains in Western culture and as a result, women often avoid and/or are embarrassed to discuss their sexual health with their health care professionals (HCPs). Moreover, midlife women are typically unaware or have misconceptions about conditions that may adversely impact their sexual life, such as genitourinary syndrome of menopause and hypoactive sexual desire disorder. Without understanding there may be underlying medical conditions, there is also a lack of awareness that safe and effective treatments are available. Lack of training, tools, time, and limited treatment options impede HCPs from providing women with necessary sexual health support. Educating women, training HCPs, and providing communication tools to HCPs can facilitate effective dialog between patients and HCPs. More specifically, HCPs can be trained to initiate and maintain a sexual health conversation in a manner that is comfortable for women to convey sexual health needs and concerns, and for HCPs to correctly identify, diagnose, and treat the sexual problems of their female patients.

***Conclusions:*** Solutions exist to address the barriers currently impeding patient–clinician interactions around sexual health.

## Introduction

The concept of sexual health has evolved significantly since the definition offered by the World Health Organization in 1975.^1^ Although different definitions of the term continue to exist today, the general principles of “autonomy and pleasure and lack of coercion and lack of violence and a positive contribution to one's overall well-being,” tend to thread across most definitions, offering useful guideposts for clinicians and researchers alike.^2,3^ Specifically, the assertion that sexual health is a vital sign for overall health is a foundational principle guiding the issues raised in this article. Although “sexual health” may include issues related to having sex, such as contraception and sexually transmitted infections, this article will use the term specifically to refer to healthy female sexual function. Despite the previous assertion, many adult women in the United States today continue to go without the necessary medical counseling, support, and/or care to fully address their sexual health needs and concerns. This is especially true for women experiencing symptoms such as painful sex, decreased desire or pleasure, and/or orgasmic disorders.

Although adult women of all ages can experience distressing sexual health-related conditions or concerns, women through the menopausal transition and beyond tend to experience these conditions with greater frequency.^4,5^ Genitourinary syndrome of menopause (GSM), which includes vulvar and vaginal atrophy,^6^ is a highly prevalent medical condition associated with menopause^7–9^; typical symptoms include sexual pain, genital dryness, vaginal irritation/itch, and/or postcoital bleeding. These symptoms are often chronic, persisting through menopause,^10–12^ and have the potential to interfere with a woman's sexual activity, intimate relationships, lifestyle, and self-esteem.^7,8,13–15^ In fact, symptoms of GSM have been found to impact women's quality of life to a similar extent as arthritis, chronic obstructive pulmonary disease, asthma, or irritable bowel syndrome.^16^

**FIG. 1.**
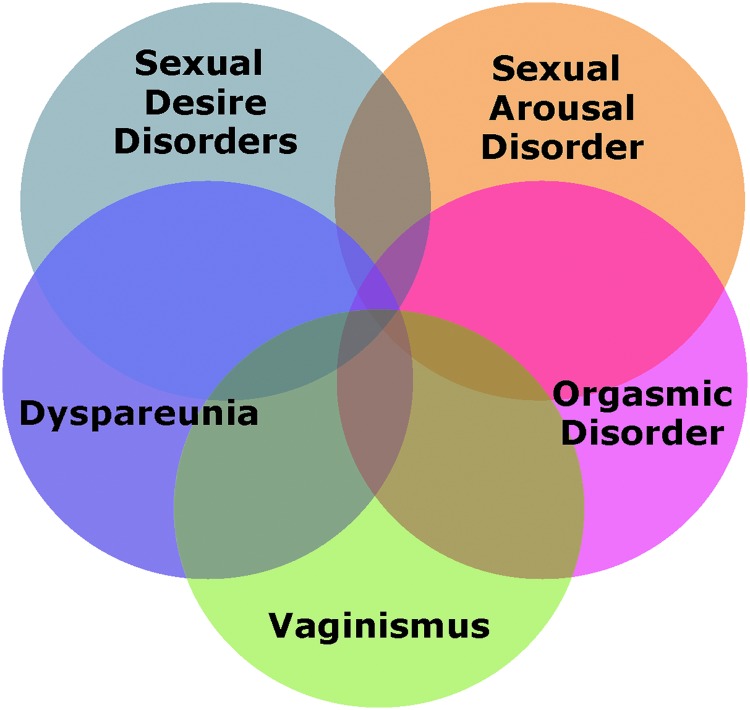
Overlap of female sexual disorders.

Female sexual dysfunction (FSD), which refers to a number of sexual dysfunctions (*e.g.*, loss of desire, decreased arousal, inability to reach orgasm and pain with sexual activity) that commonly overlap (Fig. 1), may be exacerbated by changes that occur around the menopausal transition for women. A hallmark criteria for all FSDs is personal distress,^17^ resulting in a reduced quality of life for affected women. For example, women with hypoactive sexual desire disorder (HSDD), the most common FSD, report low scores on medically recognized instruments that measure quality of life (including the SF-36 and SF-12 Health Surveys and the EQ-5D)^18,19^ that are comparable in magnitude to people suffering from back pain or diabetes.^18^

Gaps in care for these types of sexual health concerns occur for multiple reasons. For patients, reasons include social stigma and fear of broaching the topic, low awareness of the conditions and/or availability of effective treatments, and misperceptions about known treatments. For health care professionals (HCPs), suboptimal care may be because of time constraints; lack of training about diagnostic tools and treatment options; and costs, coverage, and regulatory/policy issues. Collectively, these issues often lead to inadequate sexual health outcomes for women.

**FIG. 2.**
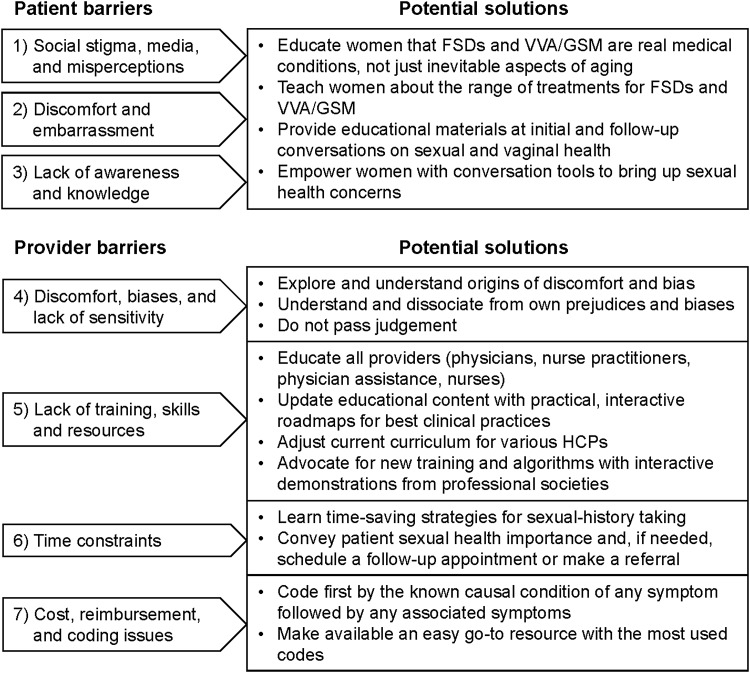
Patient-related and health care provider-related barriers aligned with potential solutions.

The purpose of this article was to explore key obstacles to better outcomes in women's sexual health and suggest a roadmap for developing solutions to these barriers. Figure 2 outlines major barriers and the possible solutions that will be discussed in this article. Although we recognize that systemic cultural and regulatory/policy changes may be necessary to address certain challenges in the long term, the recommendations and considerations in this article focus on tangible actions HCPs can take to have a meaningful impact in the near term.

## Factors Contributing to Suboptimal Sexual Health Outcomes in Adult Women

### Social stigma and conversation avoidance

Based on cultural norms and biases, conversations about sex are sometimes thought of as taboo in American society and in many other cultures worldwide. This is especially true for women, and particularly when sex is for pleasure rather than reproductive purposes. Failure to have informative discussions about sex often leads to misperceptions about sex and sexuality, including a sense that pain or lack of interest in sexual activity is inevitable and nonmodifiable, which can also lead to women not seeking the care they need. In addition, women sometimes assume that older people do not, or should not, engage in sexual activity.

Despite society not fully appreciating the importance of sexuality in older individuals, studies indicate that sexuality and/or sexual activity is important to women at all stages of adulthood, including in the postmenopausal years. The Survey of Midlife Development in the United States found that ∼60% of women older than 60 years of age were sexually active,^20^ and another survey found that 22% of married women between 70 and 79 years old are sexually active.^21^ In the AARP Survey of Midlife and Older Adults, ∼60% indicated that sex is a critical component to a good relationship^22^; and in another survey, sexual activity was positively associated with quality of life and successful aging.^23^ When assessing sexual activity in older adults, it is important to consider that sexual expression may extend beyond penile/vaginal intercourse. For many older individuals, sexuality is still important despite age-related difficulties such as erectile dysfunction (ED) or limited mobility, and for such couples, sexual activity may include other forms of intimacy or masturbation.

#### Female patients often apprehensive to discuss sex and sexual health with HCPs

Several U.S. and international surveys of women recently found that the majority of women surveyed did not discuss their sexual health-related symptoms with their HCPs, and discomfort and/or embarrassment with having this discussion was often cited as a reason for avoiding the conversation.^7,24–26^ This finding was consistent for women across different demographics, including age, sexual orientation, race/ethnicity, educational level, and relationship status.^27^

Previous negative experiences discussing sexual health with a clinician or concerns about how an HCP may react to the conversation often deter women from raising sexual health topics. Many fear that their concerns will be dismissed or considered unimportant. Furthermore, if they do raise a sexual health issue and feel it was not adequately addressed by the practitioner, women may perceive that their practitioner does not value their sexual health as a priority and/or believe that it is not amenable to treatment.^24,28,29^

Women in internationally conducted focus groups reported that when they broached the subject of sexual health symptoms, their HCPs tended to display a lack of sensitivity to these symptoms affecting their quality of life.^30^ In one survey, 59% of women believed that other people do not want to hear about their “vaginal problems”^7^; and in another, more than one-third of women who sought help for sexual health issues felt their concerns were not adequately addressed or not taken seriously enough.^25^

Women may also wrongfully fear they are alone in experiencing these kinds of symptoms or that their HCPs will view them (and their sexual health concerns) as unimportant. Women may also be concerned that there is not enough time during their appointment to address such a sensitive and multifaceted topic.

Although some women would like to discuss their sexual health with their HCP, they may not know how to bring up the subject or may be unsure of which clinician they should discuss it with. No one specialty “owns” sexual health, which can lead to patient confusion about where to best seek help. Women may also be unsure of how to articulate their symptoms and may downplay the extent of their symptoms if they are uncomfortable discussing them.

Numerous surveys highlight that women tend to expect their HCPs to initiate dialog around sexual health. In the REal Women's VIews of Treatment Options for Menopausal Vaginal ChangEs (REVIVE) survey, 40% of women expected HCPs to initiate a conversation about their symptoms^26^; and in another recent survey, two-thirds of menopausal women agreed that HCPs should inquire regularly about sexual health.^27^

#### HCPs may forego initiating conversation on sexual health

Some HCPs do not initiate the conversation on sexual health because of a lack of confidence, personal discomfort, or a sense of discomfort on the part of the patient.^29,30^ Time constraints for office visits may also play a role in HCP hesitation to raise sexual health concerns. In addition, HCPs may take an inconsistent and/or avoidant approach to address the sexual health concerns of their older female patients, which may be because of an assumption that women's sexual function is less relevant beyond reproductive years.^28,31^

In one survey, 73% of women said their primary care providers asked about sexual health only a few times or almost never.^27^ In the REVIVE survey, only 19% of menopausal women were asked by their HCPs about sexual health.^26^ In the Vaginal Health: Insights, Views and Attitudes (VIVA) survey, 50% of women stated their HCPs had not raised the topic of menopausal vaginal health.^8^

HCPs have self-reported on the frequency with which they discuss sexual health with patients. In a survey of OB/GYNs (obstetricians–gynecologists), two-thirds routinely asked about sexual activity, but only 40% asked about sexual concerns and 29% about sexual satisfaction.^32^ In a survey of nurses specifically caring for heart failure patients, 75% felt they had a responsibility to discuss sexual health with their patients. However, only 38% did so occasionally, 53% rarely, and 8% never assessed their patients' sexual health.^33^ Nurses cited a lack of knowledge regarding how to start the conversation as a reason for not discussing sexuality with patients.^33^

### Low awareness of sexual health conditions and/or availability of effective treatments

Women often do not think of their sexual health concerns as a medical condition, which may be a reason why they do not raise the subject with their HCPs. In particular, many women do not recognize GSM as a common consequence of menopause, especially because symptoms, including vaginal dryness and dyspareunia, often do not present until several years after they stopped experiencing menstrual cycles. Instead, many women view GSM symptoms as an inevitable part of the aging process, rather than a medical condition amenable to treatment. Many women remain unaware that GSM symptoms, including dyspareunia, may be because of menopause,^8,14,24,26,34^ and the associated decreased levels of estrogens and other sex steroids. Similarly, surveys of participants in an HSDD registry found the most frequent reason women suffering from HSDD did not seek medical help was because they believed the symptoms were just a natural part of aging or being in a long-term relationship.^35^

Lack of women's awareness of the availability of effective treatments for sexual health conditions also creates barriers. Despite the decades-long availability of effective vaginal estrogen therapies to treat GSM, the Women's EMPOWER survey found approximately two-thirds of participants were only somewhat familiar to unaware of effective treatments for their symptoms.^24^ Forty percent of participants in the aforementioned HSDD registry for women also reported delaying or foregoing professional help for their symptoms because they did not believe a treatment existed.^35^

As with patients, many HCPs also remain unaware of available and approved treatment options; and some HCPs may even believe that certain sexual health conditions are not real medical conditions, are not within their purview, or are exaggerated by pharmaceutical companies.

### Treatment misperceptions among patients

When women are aware of the availability of vaginal hormone therapy, including estrogens and prasterone (a synthetic form of dehydroepiandrosterone or DHEA), for treatment of GSM, they frequently have concerns about safety. The Women's Health Initiative (WHI), released in 2002,^36^ dramatically changed HCP and patient perceptions around the safety of estrogen use in menopause. The WHI, which studied oral conjugated estrogens (alone in hysterectomized women or with medroxyprogesterone acetate in nonhysterectomized women), has been widely criticized, most notably for the average age of enrollment being >10 years postmenopause.^36^ Since the early results from the WHI evaluating the oral standard dose of conjugated estrogens with medroxyprogesterone acetate, the “class labeling” for all estrogen-containing medications, including low-dose vaginal estrogens, has been required by the Food and Drug Administration (FDA). The North American Menopause Society (NAMS) and others have advocated for modification of labeling for low-dose vaginal estrogens based upon the minimal systemic exposure with blood levels remaining within the normal postmenopausal range and without increased risk of heart disease, stroke, blood clots, or probable dementia.^37^ However, apprehension about estrogen therapy because of fear and the unchanged boxed warning remains for many women. As a result, HCPs may find it challenging to overcome this negative perception when counseling their patients on low-dose vaginal treatment options.

Women may also receive inaccurate information about the efficacy of treatments marketed to address sexual health conditions. Unfortunately, the popularization of claims about unproven treatments is prevalent, as are negative perceptions about effective treatments. Celebrities and others in the public spotlight may confuse women by touting the supposed benefits of unproven methods, such as “vaginal rejuvenation,” while dismissing well-studied, effective therapies with longer term safety data. Of importance, the FDA recently warned not to use energy-based devices (radiofrequency or laser) that have received clearance for general gynecologic indications for vaginal “rejuvenation,” cosmetic vaginal procedures, or nonsurgical vaginal procedures to treat symptoms related to menopause, urinary incontinence, or sexual function until further clinical trial data confirms efficacy and safety for specific issues.^38^ In addition, alternative therapies, such as “bioidentical” compounded hormones and platelet-rich plasma are promoted as “natural” treatments for GSM, but are not regulated by FDA with regard to efficacy, safety, or marketing claims, in marked contrast to FDA-approved treatment options.

### Lack of HCP training, tools, and treatment options

Most HCPs, including physicians, physician assistants, nurse practitioners, and nurses, receive limited formal sexual health training. Although sexual medicine has grown substantially in the past 20 years, aspects of training continue to lag behind scientific and clinical knowledge in the field.^39^ In addition, the time devoted in medical school to sexual health and function and all of its complexities has dramatically decreased in medical schools.^40^ A 2016 review of existing and future educational needs in graduate and postgraduate medicine found that sexual medicine objectives were included in only a few residency programs.^39^ Even when these requirements were included, they were typically defined in a very general fashion (*e.g.*, family medicine guidelines call for “teaching of human sexuality”; gynecology residents receive training in contraception, infertility, menopause, high-risk sexual behavior, and specific sexual medicine skills such as sexual history taking).^39^ Of note, even training for urogynecologists focuses only on pelvic floor dysfunction. Professional societies may offer optional formal training on FSDs; in addition, continuing medical education (CME) may be sought by HCPs to address gaps in knowledge.^39^ However, some HCPs may not be motivated to seek out additional “optional” CME training, for which availability also varies.

Despite the fact that medical society guidelines and associated tools exist for screening common sexual health conditions like HSDD, HCPs may be unfamiliar with them or may fail to use them for patient diagnosis. HCPs may also not be familiar with educational and/or referral resources for their patients, which may prevent them from addressing sexual health at an office visit. Office-visit time constraints may contribute to HCP hesitating to raise sexual health concerns, especially if they have not received training and tools to help conduct these conversations in an efficient manner.

In addition, clinicians may feel they have limited therapeutic options to use to treat certain sexual health conditions. Kingsberg et al. recently provided an extensive overview of the psychosocial and pharmacologic (hormonal and nonhormonal) treatments for FSD, including those for HSDD, female sexual arousal disorder, and female orgasmic disorders, and found treatments in this area are severely lacking.^41^ There is currently one FDA-approved treatment for HSDD (flibanserin) and no approved treatments for arousal or orgasmic disorders. Although the indicated population for flibanserin in HSDD is currently limited to premenopausal women only, data showed that flibanserin improved sexual function in postmenopausal women with HSDD.^42,43^ In addition, a variety of over-the-counter lubricants and moisturizers, vaginal estrogens, and two nonestrogen therapies (oral ospemifene or vaginal prasterone) are available for GSM and/or dyspareunia, although surveys indicate that all GSM treatments are underutilized.^24,26^

Considerable public debate has ensued over a perceived gender disparity in sexual health drug development, as there have been approved treatments for men with ED for 20 years. This may be because of differences in the underlying etiologies of these conditions, the additional regulatory burden of evaluating separately pre- and postmenopausal women, or some combination thereof. Regardless, the advent of safe, effective treatment options for men suffering from ED, coupled with frequent direct-to-consumer advertising, has largely moved a previously stigmatized condition into open and acceptable dialog, with men now comfortable vocalizing their sexual health needs to their HCPs and HCPs feeling equipped with therapeutic options to address the concern. A national probability sample of 1550 women and 1455 men aged 57 − 85 years revealed that sexual dysfunction has negative concomitant outcomes and that it is more common in women (11.3% −43.3%) than men (2.9% −36.9%).^44^ In addition, the Fourth International Consultation on Sexual Medicine (2015) found that women were more likely to have multiple sexual dysfunctions than men.^45^ Given the nearly two decades that male sexual health has been a topic of frequent and comfortable public discourse, and the recognition that FSD is more prevalent, we assert that female sexual health must be similarly recognized, valued, and prioritized as a component of overall health.

### Cost, coverage, and regulatory/policy issues

Cost is a consideration for any treatment, particularly if managed care organizations and other gatekeepers of health care dollars deprioritize the relative importance of a therapeutic area, as is often the case with female sexual health. Clinicians may be reluctant to prescribe effective treatments if they suspect the cost to the patient at the pharmacy may be higher than she is willing or able to pay. As a result, they may recommend less expensive (although ineffective) alternatives, turn to unregulated compounding, or recommend over-the-counter vaginal lubricants and moisturizers (sometimes less expensive), which are effective for mild symptoms of vaginal dryness and/or dyspareunia, but do not treat the underlying vulvovaginal changes associated with decreased sex steroids that lead to dyspareunia. Moreover, given their patients' numerous and varied insurance benefits, HCPs may find it difficult to know offhand which treatments are covered by which insurance plans. Cost may also be a barrier for referrals to other clinicians for counseling and cognitive-behavior therapy for treating sexual dysfunction.

Relatedly, reimbursement of treatments for women's sexual health-related conditions is often lacking. Medicare had considered dyspareunia as a result of menopause as a sexual dysfunction and did not provide coverage for medical treatments, as has occurred with other “lifestyle” conditions. NAMS, International Society for the Study of Women's Sexual Health (ISSWSH), and the American College of Obstetricians and Gynecologists (ACOG) had advocated for its coverage. In May 2018, the Centers for Medicare and Medicaid sent clarification to NAMS, ISSWSH, ACOG, and others committed to women's health that drugs approved by the FDA for the treatment of moderate to severe dyspareunia, a symptom of vulvar and vaginal atrophy because of menopause, are not excluded from Medicare Part D under §1860D-2(e)(2)(A) of the Social Security Act.^46^

The necessity of a boxed warning for safety, currently required as part of the label for low-dose, vaginal estrogen-containing therapies to treat GSM, is a further obstacle that has been called into question by gynecologic HCPs in recent years.^37^ An oral selective estrogen receptor modulator, ospemifene was FDA approved in 2013 with a modified boxed warning; an intravaginal medication containing prasterone was FDA approved in 2016 without a boxed warning that may facilitate acceptance by patients. However, boxed warnings remain for all vaginal estrogen therapies.

Although no significant alcohol interaction with flibanserin was reported in the large pivotal efficacy and safety trials,^42,43^ in a small alcohol challenge study, 4 of 23 men (17%) experienced hypotension or syncope requiring therapeutic intervention.^47^ As a result, a Risk Evaluation and Mitigation Strategy (REMS) program for flibanserin was established. This REMS requires prescribers to become certified to prescribe (and pharmacists to dispense), which has limited access for women. In addition, the recent approval of flibanserin in Canada does not have an alcohol intake restriction as it does in the United States. At present, there is only one other product in development (bremelanotide) for treating HSDD that has been submitted to the FDA with a Prescription Drug Fee User Date of June 2019. The FDA should maintain warnings and restrictions on such medications that are based on objective criteria and not hold medications for sexual dysfunction to a different standard.

## Overcoming Barriers: Practical Recommendations to Encourage Better Clinical Interactions

As outlined previously, barriers to optimal sexual health outcomes for women are numerous, complex, and often interrelated. We recognize some problems are more solvable than others. Although we support efforts to address all obstacles, a necessary and achievable first step is to foster more open and informed dialog about sexual health between women and their HCPs. This simple but critical measure will improve women's health outcomes^48,49^ and can be accomplished in the near future. Therefore, the specific recommendations of this article address strategies to enable better HCP–patient communication with a focus on skills training for professionals and patient education and empowerment programs (Fig. 2). We believe that several barriers need to be addressed with such training and education.

### Training HCPs and facilitating communication

All HCPs (physicians, nurse practitioners, physician assistants, mental health professionals, nurses, etc.) who provide health care for women can benefit from training in the basics of female sexual health and dysfunctions in combination with communication skills training to facilitate successful, candid discussion about sexuality. More specifically, HCP training should focus on integrating knowledge about sexual health with skills for counseling patients and shared decision-making based on individual needs and goals.

#### HCP training

Knowledge acquisition about basic sexual health content and fundamental interviewing/communication skills should begin at the earliest stage of professional training, for example, in medical and professional schools. Despite the ever-growing competition for time with exponential growth in knowledge to be learned, advocacy efforts must be expanded to protect sexual health content and communication skills in core curricula. Further training (or in some cases, even initial training) can occur in residency or other postgraduate training. HCPs already in clinical practice who need basic training or are interested in enhanced training in female sexual medicine can seek out CME programs on sexual health education. Women's health and/or sexuality-related professional associations can also play an important role in creating and disseminating resources and best practices for practitioners (Table 1) on how to counsel patients on sexual health in the clinical setting.^39^

**Table 1.** Relevant Health Care Professional Resources

*Association*	*Title*
American College of Obstetricians and Gynecologists^54^	Committee Opinion 706: Sexual Health
American College of Obstetricians and Gynecologists^17^	Practice Bulletin 119: Female Sexual Dysfunction
International Society for the Study of Women's Sexual Health^59^	Process of Care for Management of Hypoactive Sexual Desire Disorder in Women
International Society for the Study of Women's Sexual Health^60^	Hypoactive Sexual Desire Disorder: International Society for the Study of Women's Sexual Health Expert Consensus Panel Review
International Consultation of Sexual Medicine^61^	Definitions of Sexual Dysfunctions in Women and Men: A Consensus Statement from the Fourth International Consultation on Sexual Medicine 2015
International Consultation of Sexual Medicine^62,63^	Toward a More Evidence-Based Nosology and Nomenclature for Female Sexual Dysfunctions: Parts I and II
North American Menopause Society^64^	Management of symptomatic vulvovaginal atrophy: 2013 position statement of The North American Menopause Society
North American Menopause Society^65^	Sexual Health website for menopausal women

Such best practices can be accomplished in almost any office visit setting. HCPs can first legitimize the importance of assessing sexual function and normalize the discussion by including it as part of the routine medical history. One suggestion to put patients at ease may be to mention at the outset that many patients have sexual health concerns or symptoms, providing an opening for them to ask if the patient has similar concerns. HCPs can use open-ended questions about sexual concerns instead of “yes/no” questions, which tend to hinder women's ability to accurately describe symptoms or concerns. In addition, open-ended inquiries give patients permission to talk about their sexual concerns. Routine discussion of sexual health allows HCPs to reassure women that some feelings and symptoms are common and legitimate. Any problems related to sexual response, including desire, arousal, orgasm, and pain can be explored, along with potential treatment options. This is an efficient model for HCPs to simultaneously educate women about normal sexual response and assess for problems with desire, arousal, orgasm, or pain.

The PLISSIT model is a helpful tool for discussing sexual health or concerns with patients.^50,51^ PLISSIT stands for permission (P), limited information (LI), specific suggestions (SS), and intensive therapy (IT), as outlined in Table 2. When conducting an interview, the Partnership (P), Empathy (E), Apology (A), Respect/Reflect/Reinforce (R), Legitimize (L), Support (S) (PEARLS) model can also provide a useful structure to facilitate open communication (Table 2).^52^

**Table 2.** Models for Open Clinician–Patient Communication about Sexual Health

*Model*	*Abbreviation*	*Meaning*	*Example*
PLISSIT^50,51^	P	Permission for the patient to discuss their sexual concerns (confirming normalcy), permission to continue doing what they are doing, OR permission to begin sexual assessment with open-ended questions	“Many of my patients have problems with their sexual health, what concerns you about your sexual health?”
	LI	Limited information provided about physiological changes	Provide information on the normal and pathologic changes that may affect sexuality
	SS	Specific suggestions for care plan based on open-ended questions	“Use of lubricant during intercourse is a helpful way to reduce pain from penetration that is a result of dryness”
	IT	Intensive therapy for sexual problems, if needed	Psychotherapy may be needed for a patient who has been sexually abused
PEARLS^52^	P	Partnership (HCP and patient in this together)	“Whatever sexual problems you are having, we can work on them together”
	E	Empathy (express understanding to the patient)	“I understand that what you are going through is very depressing”
	A	Apology (acknowledge any wrongdoing)	“I'm sorry you had to wait so long to see me”
	R	Respect/Reflect/Reinforce (acknowledge the patient's suffering, difficulties, etc.)	“That sounds like a very difficult situation”
	L	Legitimize (acknowledge the legitimacy of the complaint)	“What you complain of is a real medical condition”
	S	Support (convey the HCP will not abandon the patient)	“I will do whatever I can to help you”
			“These concerns are common and I have personally helped many women address them”

HCP, health care professional.

Essential clinical competencies for communication about sexual health concerns include the ability to initiate a direct and concise conversation about sexual health in a space that ensures privacy and comfort. For example, the HCP and patient should both be seated face-to-face with the patient clothed. The HCP should complete a brief sexual health history, discuss any concerns, and close the conversation with shared decision-making and a suggestion for a follow-up appointment to further assess and treat. Alternatively, the HCP can provide a referral to a sexual medicine expert or sex therapist who can address more complex concerns. The International Urogynecological Association (IUGA) and International Continence Society (ICS) stress that sexual concerns should be addressed routinely and in a recent report suggested an educational process similar to the above to be used in women with pelvic floor dysfunction, given that most pelvic floor dysfunctions are believed to negatively affect sexual health.^53^ The ACOG Committee Opinion on sexual health, meant to increase awareness of the importance of addressing women's sexual health in routine practice, provides a listing of questions to be utilized during sexual history taking.^54^ We advocate that all HCPs of any specialty should be able to initially address sexual health issues, or if not comfortable doing so, have a streamlined, care-path referral in place as part of their routine practice.

As noted in the PLISSIT model, beyond providing basic information and suggestions, many HCPS may still want to refer a patient to qualified sexuality specialists. Appropriate sexual health referrals could apply to HCPs in any specialty area. For example, a neurologist treating a patient with multiple sclerosis could discuss sexual health with her patients, and if lacking in expertise, be able to provide them with some appropriate HCP recommendations. Professional associations could help HCPs direct women to sexual health specialists by having a mechanism for HCPs to identify appropriate professionals by location and areas of expertise.^55^

Increasing HCPs' familiarity with appropriate *International Classification of Diseases* (*ICD*) codes for FSDs (Table 3) will help address the barrier to care caused by HCPs avoidance of assessing sexual concerns because of lack of awareness that there are corresponding billing codes they can easily use for ensuring insurance coverage and payment of visits and treatments for patients with sexual health-related concerns. FSDs listed in the *Diagnostic and Statistical Manual of Mental Disorder*, Fifth Edition (*DSM-5*) are included in Mental, Behavioral and Neurodevelopmental Disorders *ICD-10* codes that begin with the letter “F.” “F” codes are appropriate for mental and behavioral health care diagnoses. HCPs in the clinical setting should consider correlating symptom codes, which may be more appropriate when patients present with symptoms of FSD, before an actual diagnosis is made. Various other codes may also be considered to support the diagnostic workup of FSD and referral to pelvic floor physical therapy. The known causal condition of any symptom should be coded first, followed by any associated symptoms of the condition. For example, low libido because of pain with intercourse would be coded first by pain with intercourse and then for low libido (Table 3). HCPs should always check with a billing and coding specialist for the most recent information.

**Table 3.** *International Classification of Diseases*, Tenth Revision (*ICD-10*) and Current Procedural Terminology Codes^a^ for Female Sexual and Pelvic Floor Dysfunctions and Genitourinary Syndrome of Menopause

*Diagnosis or reason*	*Code*
Female sexual dysfunction (*DSM-IV* diagnosis)	
Hypoactive sexual desire disorder	F52.0
Sexual aversion disorder	F52.1
Female sexual arousal disorder	F52.22
Female orgasmic disorder	F52.31
Dyspareunia not due to a substance or known physiological condition	F52.6
Vaginismus not due to a substance or known physiological condition	F52.5
Interest and arousal disorders	
Low libido	R68.82
Vaginal dryness	N89.8
Vaginal dryness, menopausal	N95.1
Persistent genital arousal disorder	N94.89
Pain disorders	
Dyspareunia	N94.1
Vaginismus	N94.2
Vulvodynia (other, unspecified)	N94.818/819
Vulvar vestibulitis	N94.810
Atrophic vaginitis/genitourinary syndrome of menopause	N95.2
Pelvic/perineal pain	R10.2
Pain in joint, pelvic region, and thigh	M25.559
Additional codes that may support physical therapy	
Weak pelvic floor muscles	N81.89
Muscular wasting and disuse atrophy	M62.50
Generalized muscle weakness	M62.81
High tone pelvic floor dysfunction	N94.89
Spasm of muscle	M62.838
Lumbago	M54.5
Unspecified disorder of muscle, ligament, or fascia	M62.9
Additional codes that may support bloodwork	
Symptomatic menopausal states	N95.1
Premenstrual tension syndromes	N94.3
Fatigue	R53.83
Apathy	R45.3
Low testosterone in female	E34.9
Emotional lability	R45.86
Weight gain	R63.5
Common CPT codes in sexual medicine	
Wet prep	87210
Vaginal pH	83986
Genital culture (specimen handling)	99000
Trigger point injections	
<3 muscles	20552
3+ muscles	20553
Colposcopy/vulvoscopy	
Vulva	56820
Vulva with biopsy	56821
Biopsy	
Vulva/perineum	56605
Additional lesions of vulva/perineum	56606
Vagina	57100
Extensive biopsy of vagina requiring suturing	57105
Perineometry (biofeedback)	90911
Pelvic floor physical therapy assessment	
Low complexity	97161
Moderate complexity	97162
High complexity	97163
Fractional CO_2_ laser therapy^b^	58999

^a^ Regarding coding: Every reasonable effort has been made to ensure the accuracy of the information within this article. However, the ultimate responsibility for compliance with Medicare rules and regulations lies within the provider of services.

^b^ Unlisted procedure female genital system (nonobstetric).

*DSM-IV*, *Diagnostic and Statistical Manual of Mental Disorder*, Fourth Edition.

#### Establishing and maintaining patient trust

A theory-based qualitative study of women older than 50 years of age found that women who wanted to communicate with their HCPs about sexual health would only do so if they felt comfortable and trusted their provider.^56^ HCPs can build rapport with a patient by asking questions about sexual health as comfortably as they would ask other health questions, by not rushing them through the discussion, by remaining nonjudgmental, and by assuring them that what is discussed will remain confidential^29,56^ and that what they are experiencing is normal. Specifically, a matter-of-fact attitude,^29^ explanation that these are common conditions, and reassurance that they are treatable may help women relax when discussing these topics.

HCPs may initially feel confused by the common overlap of sexual concerns (*i.e.*, problems with desire, arousal, and orgasm [Fig. 1]). However, simple inquiry regarding which problem developed first or is most distressing typically informs treatment decisions. Once a sexual health concern has been identified and addressed, HCPs should continue to check in with patients and discuss issues of sexual health on subsequent visits. At any stage, conversations about sexual health may be enhanced by providing credible and accurate educational information and resources to women.

Guidance should be given to HCPs to begin conversations on sexual health without any assumptions about sexual activity, sexual orientation, relationship status, or any other topics that would impede a connection with the patient,^48^ or could make them feel judged or ashamed. It is also important for HCPs to be sensitive and not pass judgment on women who may be engaged in sexual activity with women, men, or both, or self stimulation, and not assume that they are in a monogamous heterosexual relationship.^57^

Developing and using language that puts women at ease is also helpful in building trust. For example, postmenopausal women do not consider “vaginal atrophy” a suitable term for vaginal discomfort,^8,14^ and they may be less likely to discuss it if this language is used. Using plain language, props, and/or illustrative aids, and describing sexual anatomy and physiology in simple terms may help to make the explanations clearer. For instance, clinicians may want to use the terms “low libido” or “low desire” rather than HSDD. Many HCPs also find it beneficial to provide patients with a hand mirror to show the external genital changes that have occurred, or advise that their vaginal pH is alkalotic instead of acidic, both of which may also help make the patient and HCP feel more comfortable.

In addition, HCPs should be encouraged to explore the origins of their discomfort,^31^ and to understand their own biases through unconscious bias and empathy training exercises. They should also be aware of how their own personal views about sexuality could impact their interaction with patients, so they can dissociate these personal biases and views from their discussions with patients.^29^ A small survey of women 40–75 years old found that women were more likely to discuss sexual health when the HCP did not make assumptions and appeared nonjudgmental.^57^

#### Overcoming time constraints

To overcome time constraint issues, HCPs can be trained on time-saving strategies that can be implemented at various stages of the initial office visit. A practical suggestion is to first take a sexual history using open-ended questions (as opposed to “yes/no” questions), which can convey a great deal of information in a relatively short amount of time (≤5 minutes). In addition, HCPs can use preformulated questionnaires, such as the decreased sexual desire screener, meant to facilitate the diagnosis of HSDD when clinician–patient time is limited.^58^ Clinicians could consider questionnaires before seeing patients in the office to save time; however, follow-up on the questionnaire during in-office, clinician–patient interaction is critical, as not all patients are comfortable with providing written documentation on this sensitive subject. HCPs should also address the most important topics that can be covered in a limited amount of time. Instead of feeling pressured to cover it all in one visit, they should convey to patients that their sexual health is important and encourage a follow-up appointment that focuses solely on sexual health concerns. Providing written information for patients to review may help facilitate a patient's return visit.

#### Making the most of medical intake

In addition to sexual health being addressed in routine medical history, other strategies to assess sexual health concerns include adding prompts in the electronic medical record or including sexual health questions on medical intake forms.^57^ Although some women may be concerned that inclusion of such information in an electronic record violates their privacy, they should be reassured that such information is kept secure and confidential, and only accessible by their health care providers. A system similar to that in behavioral health would be useful in which only HCPs who will be involved with the sexual issue have access to that aspect of the health record. In addition, clinicians can ask specific questions about phases of the sexual response (desire, arousal, orgasm) and pain to provide examples of the level of detail that they would like the patient to address.

Professional societies could develop creative new tools for HCPs to use in practice such as (1) clinical practice guidelines for diagnosis, management, and treatment, (2) summaries of well-conducted clinical trials, (3) ice-breaker videos, or (4) phrases to help prompt clinicians to initiate conversations that work. Clinical study summaries for HCPs can quickly summarize the main points of studies, conveying the most important data supporting the management and treatment of pertinent sexual health issues. Ice breakers and conversation prompts can offer tangible examples to help clinicians successfully broach sexual health topics with their patients.

### Educating and empowering patients

In addition to offering education to HCPs, women themselves should be better educated and empowered to discuss sexual health. Educational tools explaining normal anatomy, biological and etiological factors, and sexual response, as well as possible causes of dysfunction, should clear up misconceptions and emphasize that sexual health-related conditions are common, real, and sometimes, as in the case of GSM, chronic and progressive. Using a hand-held mirror as standard of care could allow women to see and be empowered about their own anatomy. Credible, easy-to-understand information should be easily available in person at the clinician's office and online. Having easily available educational materials in the office also demonstrates a HCP's comfort and awareness of sexual health treatments. In fact, a recent international survey found that nearly half of menopausal women with vaginal discomfort would have liked information or a pamphlet on the topic to help decide whether to consult with their physician about it.^7^

Most importantly, better information, resources, and tools can help women to believe that sexual health is a critical part of their overall health and well-being and should be regarded with importance similar to other aspects of health. When patients are empowered this way, they may feel more competent to manage their own health and may be more likely to follow through with treatment.^55^

## Concluding Remarks

Sexual health is as important as any other facet of health and should receive the same level of attention. The opportunity for women to freely and fully discuss their sexual health needs, concerns, and potential treatment options with a trusted HCP is critical for improving sexual health outcomes. Despite the role of sexual health as a vital sign for overall health and quality of life, the topic is unfortunately too often left unaddressed by both HCPs and female patients. Effective therapy is available. Many cultural, educational, and practical barriers contribute to the substantial under-recognition and under-treatment of sexual health-related conditions affecting women. Enhanced HCP training and support for better-guided conversations with patients and greater patient education and confidence are important starting points for developing necessary solutions to ensure optimal sexual health outcomes in women.
